# Orthotopic Liver Transplantation: Is There a Risk for *Listeria monocytogenes* Infection?

**DOI:** 10.1155/2010/901894

**Published:** 2010-03-11

**Authors:** Urs Ehehalt, Stefan Schmiedel, Ansgar W. Lohse

**Affiliations:** Medical Department I (Gastroenterology, Hepatology, Infectious Diseases), University Medical Center Hamburg-Eppendorf, Martinistr. 52, 20246 Hamburg, Germany

## Abstract

Immunosuppression of any kind is a known risk factor for infection with *Listeria monocytogenes* (*L. monocytogenes*). Particularly, patients with impaired liver function are at increased risk of developing an aggravated course of infection with this bacterial pathogen (see Nolla-Salas et al.; 2002 and Cabellos et al.; 2008). It is a well-known pathogen in immunocompromised patients, but has only seldom been reported following orthotopic liver transplantation. Invasion of the central nervous system presenting as meningitis or meningoencephalitis and bacteremia are the principal clinical manifestations of listerial infections (see Brouwer et al.; 2006). We present an account of a case of a patient who developed *L. monocytogenes* meningitis during the early period after liver transplantation.

## 1. Case Report

We report the case of a 57-year old man diagnosed with liver cirrhosis Child-Turcotte-Pugh C resulting from chronichepatitis C. He underwent liver transplantation in June 2007, at a transplantation center in Germany. The operation itself was uneventful and no complications were reported after surgery. Furthermore the preexistent hepato-renal syndrome resolved after transplantation. His immunosuppressive medication included tacrolimus (2 × 4 mg), mycophenolate-mofetil (2 × 1 g) and prednisolone (10 mg).

In August 2007 the patient was admitted to our hospital because of impaired consciousness since the previous day. In our emergency ward elevated body temperature was noticed and shortly after admittance the patient suffered a focal epileptic seizure accompanied by worsening of the mental status and the respiratory situation. He became unconscious and had to be transferred to our intensive care unit. For further evaluation a lumbar puncture was performed. The laboratory-chemical analysis indicated bacterial meningitis (see Table 1). In the microscopical analysis gram-positive rods were observed. A parenteral antibiotic therapy with ampicillin (4 g three times daily) and gentamicin (5 mg/kg) was initiated. In the following days, *L. monocytogenes* grew in culture of cerebrospinal fluid (CSF). The patient responded well to the above-mentioned therapy. The elevated laboratory parameters of inflammation (CRP on admission: 234 mg/l, CRP hospital day 6: 44 mg/l) normalized and the clinical status improved. In a followup lumbar puncture on day 6 of the clinical illness the cell count was normalizing. The patient was transferred to the normal ward after his clinical situation improved. He was discharged on the 25th day following admittance. Before the infection occurred there was no exposure to potentially contaminated food known.

## 2. Discussion

*Listeria monocytogenes* is a ubiquitous pathogen in the environment, capable of causing human and animal infection. Although uncommon in humans, it causes illness in sporadic and epidemic forms throughout the world. The pathogen is a facultative intracellular, aerobic, facultatively anaerobic gram-positive rod [1]. The organism is normally found in soil and decaying vegetable material. Listeria is exceptionally resistant to environmental conditions and able to grow in temperatures ranging from 4°C to 37°C. It is most often transmitted to humans via contaminated foods like milk, cheese, undercooked meat, or uncooked vegetables. The pathogen has also been isolated from asymptomatic humans. About one to five percent of healthy individuals are fecal excretors of Listeria species [2]. The Center of Disease Control and Prevention (CDC) declares that in the United States an estimated 2,500 persons become seriously ill with listeriosis each year; of these 500 die. 

After the introduction of the Haemophilus influenzae type b conjugate vaccine in the USA, meningitis caused by *L. monocytogenes* became the fourth most common cause of bacterial meningitis, following *Streptococcus pneumoniae, Neisseria meningitides* and group B streptococcus [3].

Most of the systemic invasive infections with Listeria are in individuals with one or more predisposing conditions. The risk factors are mainly pregnancy, corticosteroid therapy, other immunosuppressive therapy, and age. In a study from Finland seventy four cases of systemic listeriosis were analysed. Of these patients, 66% had an underlying disease, most commonly malignancy, diabetes mellitus, or renal transplantation, and 43% had received immunosuppressive therapy within one week before onset of listeriosis. Bacteremia and central nervous system infections were the most common clinical entities; both accounted for 43% of cases [4]. In a review analysing Listeria-meningitis-meningoencephalitis outside of pregnancy, hematologic malignancy and kidney transplantation were the leading predisposing factors. But in 36% of patients no underlying diseases were detectable. Listeriosis occurred throughout life, with a higher incidence before the age of 3 and after the age of 45–50 years [5]. Thirteen cases of spontaneous bacterial peritonitis due to *L. monocytogenes* in individuals with cirrhosis suggest that liver cirrhosis is also a significant risk factor for listeriosis [6]. 

According to a Spanish study bacterial meningitis in cirrhotic patients was associated with a high mortality and a large number of complications; a case fatality rate of 53.1% was observed. Of the classic pathogens, *L. monocytogenes* was more prevalent than in other immunocompromised patients. The authors suggest that this could be the result of the easy translocation of the organism into the bloodstream from the gastrointestinal tract [7]. According to a review of the literature, it seems that liver disease is not the only predisposing factor of infection with Listeria: liver transplantation is also associated with an elevated risk of infection with this bacterial pathogen. The authors collected thirteen cases of listeriosis following liver transplantation, in which four occurrences of meningitis were reported [8–21]. The case report itself referred to a case of a patient with central nervous system involvement following orthotopic liver transplantation; *L. monocytogenes* was grown in culture from the CSF and blood [8]. In another case report a 29-year old man diagnosed with autoimmune hepatitis suffered bacteremia and peritonitis in the early postoperative period after cadaveric liver transplantation [22]. Data from the literature show that listeriosis can present within days to years after transplantation [9, 10]. Nosocomial acquisition in the present case is unlikely because there were no further cases of listeriosis in our hospital at the same time. There was also no evidence that the transmission was caused by the donor. We speculate that the source of infection is through the intestinal tract following previous enteric colonization probably after ingestion of contaminated food products.

Even though there are still no satisfactory data regarding the incidence of Listeria infection following liver transplantation it seems that patients after transplantation suffer a greater risk to develop an aggravated course of infection with this pathogen. Including this case there are 16 reported cases of listeriosis after liver transplantation (see Table 2). 

The clinical signs of meningitis caused by Listeria include fever, headache and altered mental status and the signs of Listeria meningitis do not for the most part differ from those found in patients with community-acquired non-listerial bacterial meningitis [23]. All of the typical symptoms were present in 43% of the patients. Almost all patients presented with at least two of the four classical symptoms, headache, fever, neck stiffness, and altered mental status. The above-mentioned symptoms of bacterial meningitis were reported equally in meningitis patients due to other causes [24]. On the contrary in some previous reports an atypical course of meningitis was reported. In a study by Mylonakis et al., patients with Listeria infection had a significantly lower incidence of meningeal signs compared with patients with acute meningitis due to other bacterial pathogens. The CSF profile was significantly less likely to have a high cell count or a high protein concentration [5]. The course of meningitis ranges from mild illness with fever and changes of mental status to a fulminant course with coma. A subacute presentation with cranial nerve palsies, lymphocytic pleocytosis, elevated CSF protein and low glucose may mimic tuberculous or fungal meningitis. Additionally focal signs can be present. Beside cranial nerve abnormalities, there may be ataxia, tremors, hemiplegia and deafness. In the later course seizures may occur [5]. In a large proportion of patients, symptoms may be present for more than 4 days. In the literature, meningitis caused by Listeria is characterized by high case-fatality rates (24%–62%) due to the occurrence in the elderly and the immunocompromised [25]. A recent prospective study demonstrates a mortality rate of 17% [23].

We present a case of Listeria meningitis shortly after orthotopic liver transplantation. Because of the low incidence and the high mortality rate of Listeria infection, physicians must be aware of this pathogen most notably in immunocompromised patients, particularly because the therapy of Listeria meningitis diverges from the “normal” regime used for other bacterial pathogens causing meningitis. Third-generation cephalosporins are the beta-lactams of choice in the empiric treatment of meningitis. These drugs have potent activity against all the major pathogens of bacterial meningitis with the notable exception of *L. monocytogenes* since Listeria has an innate resistance to cephalosporin antibiotics. The treatment of choice against Listeria has traditionally been ampicillin (2 g every four hours) since resistance to this drug is rare [26]. Some authors recommend the addition of gentamicin to ampicillin treatment on the basis of the synergistic effect observed in vitro in animal models, but gentamicin is poor in penetrating the intracellular compartment and the CSF and is a well-recognized nephrotoxic agent. Ampicillin is given for two to four weeks in immunocompetent patients and for four to eight weeks in immunocompromised patients. Gentamicin is given for 10 to 14 days until the patient improves; in poor responders, it can be given for up to three weeks.

## Figures and Tables

**Table 1:** Cerebrospinal fluid analysis.

	Reference range, adults	On admission	Hospital day 6
Total cell count (per mm^3^)	0–5	1220	724
Proteine (mg/dl)	5–55	519	311
Glucose (mg/dl)	50–57	36	101
Lactate (mmol/l)		16.6	4.7

**Table 2:** Cases of listeria infection following liver transplantation.

Age (yr)	Sex	Time post LTX	Clinical presentation	Treatment	Outcome	Ref
53	M	5 d	Meningitis with headache, mental decline	Ampicillin	Survived	[8]
66	F	32 mo	Fever, hypotension, bacteremia	Ampicillin	Survived	[9]
39	F	7 d	Fever, abdominal pain, bacteremia	Ampicillin	Survived	[10]
66	F	32 mo	Fever, right flank pain, anorexia, bacteremia	Ampicillin	Survived	[11]
39	F	7 d	Fever, abdominal pain, bacteremia	Ampicillin	Survived	[12]
55	F	4 mo	Fever, abdominal pain, bacteremia	TMP-SMX	Survived	[13]
57	F	20 mo	Bacteremia, hepatitis	Ampicillin	Survived	[14]
56	F	8 mo	Bacteremia, hepatitis	Ampicillin, Gentamicin	Survived	[15]
41	F	10 mo	Endocarditis, bacteremia, hepatitis, pulmonary emboli	Ampicillin, Gentamicin	Survived	[16]
47	F	NR	Peritonitis, bacteremia	Ampicillin, Amikacin	Survived	[17]
11 mo	M	7 d	Meningitis, epididymitis, orchitis	Ampicillin	Survived	[18]
NR	NR	14 d	Meningitis	NR	Survived	[19]
67	F	21 d	Meningitis	Ampicillin	Died	[20]
13	F	4 mo	Meningitis	Ampicllin, Tetracycline	Died	[21]
29	M	4 d	Fever, abdominal pain, cholestasis	Ampicillin	Survived	[22]

Abbreviations: TMP-SMX: trimethoprim-sulfamethoxazole; NR: not reported.
